# Virus Infection Impairs Fungal Response to Stress: Effect of Salt

**DOI:** 10.3390/v15030718

**Published:** 2023-03-10

**Authors:** David A. Stevens, Ioly Kotta-Loizou, Marife Martinez, Robert H. A. Coutts, Gabriele Sass

**Affiliations:** 1 California Institute for Medical Research, San Jose, CA 95128, USA; 2 Division of Infectious Diseases & Geographic Medicine, Stanford University, Stanford, CA 94305, USA; 3 Department of Life Sciences, Faculty of Natural Sciences, Imperial College London, South Kensington Campus, London SW7 2AZ, UK; 4 Department of Clinical, Pharmaceutical and Biological Science, School of Life and Medical Sciences, University of Hertfordshire, College Lane Campus, Hatfield AL10 9AB, UK

**Keywords:** *Aspergillus fumigatus*, polymycovirus, salt stress

## Abstract

Infection with Aspergillus fumigatus polymycovirus 1 (AfuPmV-1) weakens the resistance of biofilms of common *A. fumigatus* reference strain Af293 in intermicrobial competition with *Pseudomonas aeruginosa*, and sensitizes *A. fumigatus* for antifungal effects of nikkomycin Z. We compared the sensitivity of two virus-infected (VI) and one virus-free (VF) Af293 strains to hypertonic salt. Salt stress impairs the growth of VI and VF at all times; VF control growth always exceeds VI, and VF growth in salt always exceeds VI. Since VF growth exceeds VI in the presence and absence of salt, we also examined growth in salt as a percentage of control growth. Initially, as a percentage of control, VI exceeded VF, but at 120 h VF began to exceed VI consistently even by this measure; thus, at that time the growth of VF in salt surges in relation to control growth, or, alternatively, its growth in salt persists compared to the relative inhibition of VI. In summary, virus infection impairs the response of *A. fumigatus* to several different stresses, including hypertonic salt.

## 1. Introduction

Fungi, similar to all living organisms, may be infected by viruses known as mycoviruses; however, there are few data on the effects of viral infections on the fungal host physiology. Most studies have failed to demonstrate any effects, contributing to the notion that mycoviruses are cryptic, while a few have shown effects on traits such as growth, sporulation, virulence and drug resistance. The current understanding of *Aspergillus* viruses, and the complexity of their effects, has been recently reviewed elsewhere in detail [1].

Aspergillus fumigatus polymycovirus-1 (AfuPmV-1) [2] is the prototype of a novel family *Polymycoviridae*. Members of the family *Polymycoviridae* and its sole genus *Polymycovirus* have multi-segmented double-stranded (ds) RNA genomes, whereas most lack a conventional capsid structure [3]. In the light of the medical significance of *Aspergillus fumigatus* (Af), which is the main pathogen causing aspergillosis in immunocompromised individuals, investigating the effects of AfuPmV-1 infection may provide insight into alternative approaches to combat Af.

In previous studies, we showed that AfuPmV-1 impairs Af in intermicrobial competition, largely in competition for iron with the pathogenic bacterium *Pseudomonas aeruginosa*, but also against effects of bacterial volatile organic molecules [4]. The former defect was related to the temporal production of Af siderophores responsible for iron uptake and storage [5]. We now studied the effect of viral infection on another stress, outside of intermicrobial competition: hypertonic salt.

## 2. Materials and Methods

Fully sequenced reference strain Af293 [6] was infected with AfuPmV-1, and an isolate was maintained in our laboratories for over 10 years, documented to be infected with the virus [2], and here relabeled strain 18–95 for purposes of this report. It was cured of infection with cycloheximide, as described [2], giving us the isogenic virus-infected Af293 isolate (18–95) and the now virus-free (18–42) strain for studies. Strain 18–95 was also reinfected with the virus, to produce strain 19–40 [2]. Strain 19–40 provides another control, guarding against the chance that the process of curing the fungus of virus could have also altered fungal cell processes.

Agar plates were inoculated with the strains under study, and when conidiation had occurred conidia were harvested, as previously described [4], to produce 10^6^ conidia/mL of inocula, and 0.01 mL of this was added to 6 mm paper discs on 4 study plates. RPMI 1640 agar plates were made using 100 × 15 mm Petri dishes; Bacto Agar (Carolina Biological Supply Co., Burlington, NC, USA) 3.75 g and 75 mL de-ionized water were autoclaved and then allowed to cool. RPMI 1640 medium, 225 mL, prewarmed in a water bath at 50 °C was added. To produce high salt agar, NaCl 14.03 g was added to the RPMI 1640 medium. The RPMI medium, with or without NaCl, was added to the Bacto agar mixture, and 25 mL per plate poured.

Growth (area, calculated from the formula πr^2^) of the isogenic strains on RPMI1640 agar in the presence and absence (control) of 0.8 M NaCl (final concentration) was compared in several experiments (4 technical replicates/experiment), over 48–144 h. The salt concentration in this study was selected on the basis of preliminary experiments, selecting a concentration showing a definite growth inhibition of strains.

Results were analyzed using Student’s t test. All data in this study are expressed as a mean ± SD.

**Figure 1:**
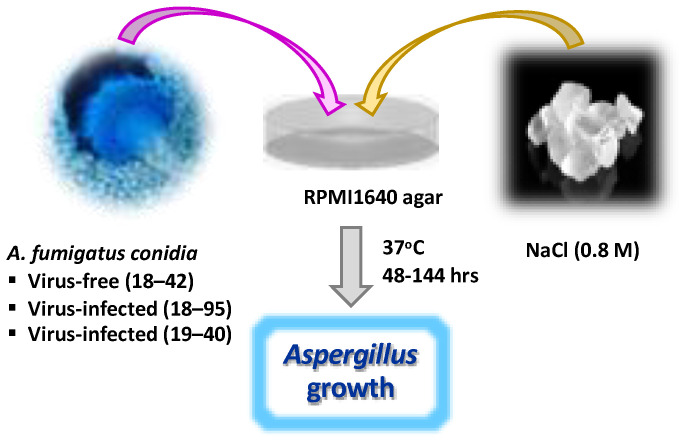
Diagram of studies. Af strains are grown on RPMI1640 agar with or without high salt at 37 °C, and measurements of growth area made daily (48–144 h).

A diagram of the experimental procedure is given in Figure 1.

## 3. Results

### 3.1. Salt Stress Impairs Aspergillus Growth

**Figure 2:**
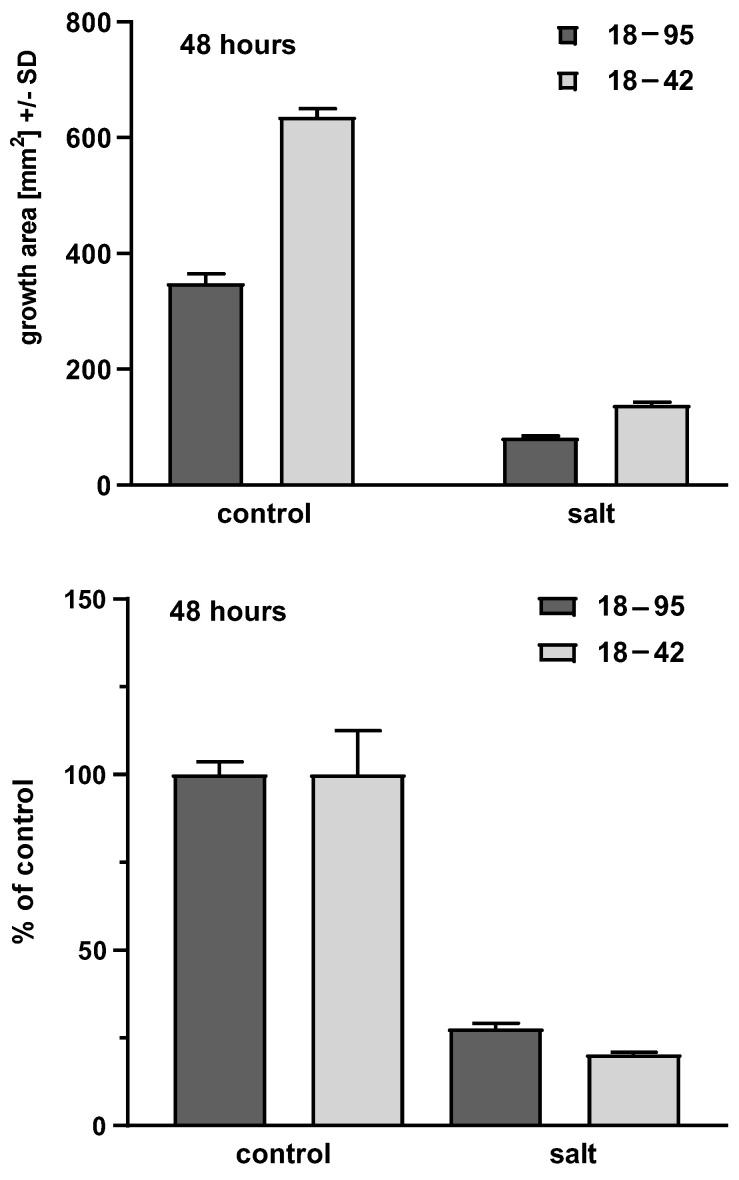
Representative areas at 48 h. Experiment 4, four technical replicates for each bar. Top part, growth areas of the virus-free (18–42) (lighter gray bars) and virus-infected (18–95) (darker gray bars) isogenic A. fumigatus strains. Left 2 bars, control growth (C) on RPMI1640 agar; right 2 bars, growth in the presence of salt. Control growth for 18–42 is superior to 18–95 (*p* < 0.0001); growth in salt is superior for 18–42 compared to 18–95 (*p* < 0.0001). Salt inhibits growth of both strains in comparison to their controls (*p* < 0.0001). Bottom part, growth in salt as percent of control growth. Shading of bars is the same. At this time, by this measure, 18–95 growth is superior to 18–42 (*p* < 0.002).

**Figure 3:**
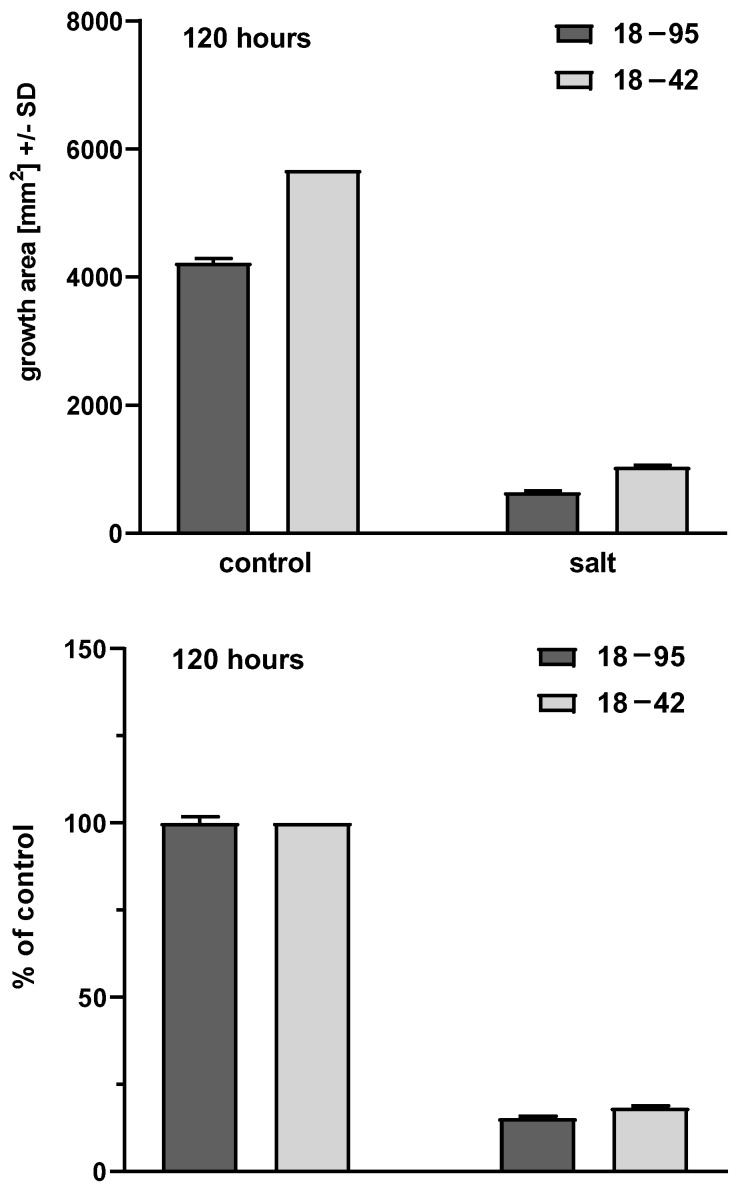
Representative areas at 120 h. Experiment 4, four technical replicates for each bar. Shading of bars is the same as Figure 2. Top part, growth areas of the virus-free (18–42) and virus-infected (18–95) isogenic A. fumigatus strains. Left 2 bars, control growth (C) on RPMI1640 agar; right 2 bars, growth in the presence of salt. Control growth for 18–42 is superior to 18–95, (*p* < 0.0001); growth in salt is superior for 18–42 compared to 18–95 (*p* < 0.0001). Salt inhibits growth of both strains in comparison to their controls (*p* < 0.0001). Bottom part, growth in salt as percent of control growth. At this time, by this measure, 18–42 growth is superior to 18–59, not only in absolute terms, but also as a percentage of control growth (*p* = 0.003). In multiple experiments, 120 h was a transition time and observations at later times (e.g., 144 h) indicated 18–42 growth in salt was always superior to 18–59 at those times, in all experiments, not only in absolute terms (as in every time point studied) but also as a percentage of control growth, as illustrated here for 120 h.

NaCl 0.8 M impaired Aspergillus growth. In five of five experiments, this was demonstrated at 48, 72, 96, 120 and 144 h, for strains 18–95 (virus-infected) and 18–42 (virus-free) (*p* < 0.0001, at all times, control growth vs. growth on salt agar). Examples of this can be seen in the top half of Figure 2 and Figure 3, comparing the virus-free and virus-infected strains in the presence (right pair of bars) and absence (left pair of bars) of salt.

### 3.2. Growth of the Virus-Free Aspergillus in the Presence of Salt Is Superior to the Virus-Infected Isogenic Strain

In six experiments, at every time point, the growth of the virus-free strain 18–42 was superior to the virus-infected strain (18–95). This was not as pronounced at the beginning of the time course, at 48 h, when impaired radial growth could first be reliably measured, or at 72 h. With further time of incubation, the difference in favor of the virus-free Aspergillus strain was consistently significant in five of five experiments at 96 h (*p* = 0.02–<0.0001), five of five at 120 h (*p* = 0.002–<0.0001) and three of three at 144 h (*p* = 0.002–0.0002). Examples of this can be seen in the top half of Figure 2 (48 h) and Figure 3 (120 h), comparing the virus-free and virus-infected strains in the presence (right pair of bars) of salt.

### 3.3. The Virus-Free Aspergillus Strain Control Growth (Absence of Salt) Always Exceeds That of the Virus-Infected Strain

In five of five experiments, at every time point studied, virus-free Aspergillus control growth was also always greater than the virus-infected strain. This was recorded at 48 h (n = 5, *p* = 0.003–<0.0001), 72 h (n = 5, *p* = 0.0007–0.0001), 96 h (n = 5, *p* = 0.002–<0.0001), 120 h (n = 5, *p* = 0.0003–<0.0001) and 144 h (n = 3, *p* = 0.0005–<0.0001). Examples of this can be seen in the top half of Figure 2 and Figure 3, comparing the virus-free and virus-infected strains in the absence (left pair of bars) of salt.

### 3.4. Over Time, the Virus-Free Aspergillus Growth in the Presence of Salt, Relative to (i.e., as a Percent of) Control Growth, Transitions from Inferior to the Virus-Infected to Superior

Given the dramatic differences both in growth between the virus-free and virus-infected strains in the presence of salt, and the dramatic differences in the absence of salt, we examined the susceptibilities of the two strains to salt as a function of the relative inhibition compared to control growth. This reiterated physiologic differences between the strains that were time-dependent. At the beginning of growth, at 48, 72 and 96 h, the relative growth in salt (as a percent of control) of the virus-free strain was less than the virus-infected at all three time points. In three of five (48 h), three of five (72 h) and two of five (96 h) experiments, the lesser virus-free strain relative growth differences were also significant (*p* = 0.03–0.002; *p* = 0.02–0.0002; *p* = 0.02–0.002 at these three time points, respectively).

The observations at 120 h appear to indicate that approximately at that time the growth of the virus-free strain in salt consistently has a relative surge in relation to the further control growth, or, alternatively stated, its growth in salt persists compared to the relative inhibition in salt of the virus-infected strain. Now, at this time, there was no inferiority in the relative growth of the virus-free strain. In two of five experiments there was no statistically significant difference between the strains, but in three of five the virus-free growth relative to control was now also significantly superior to the virus-infected (*p* = 0.03–0.003) at 120 h. This (and the observation at 144 h, mention to follow) indicated that at about 120 h there was a transition, and small variations in inoculum, growth, temperature, or time of reading between experiments could cause this transition point to lodge on one side or the other of this 120 h break point, shifting from borderline significant differences to significant differences. We presume that recordings at less than 24 h intervals at about 120 h could have defined more precisely when the difference became consistently significantly different. By 144 h, this phenomenon was consistent in that in all three experiments the virus-free strain growth in the presence of salt, relative to control growth, was now significantly superior to the virus-infected strain (*p* = 0.02–0.0004).

Examples of comparisons of relative growth in the presence and absence of salt can be seen in the bottom half of Figure 2 and Figure 3, where control growth is normalized (left pair of bars), and the virus-free and virus-infected strains are compared for growth in the presence (right pair of bars) of salt, relative to control growth.

### 3.5. The Re-Infected Aspergillus Strain Exhibits a Phenotype Similar to the Virus-Infected Strain

**Figure 4:**
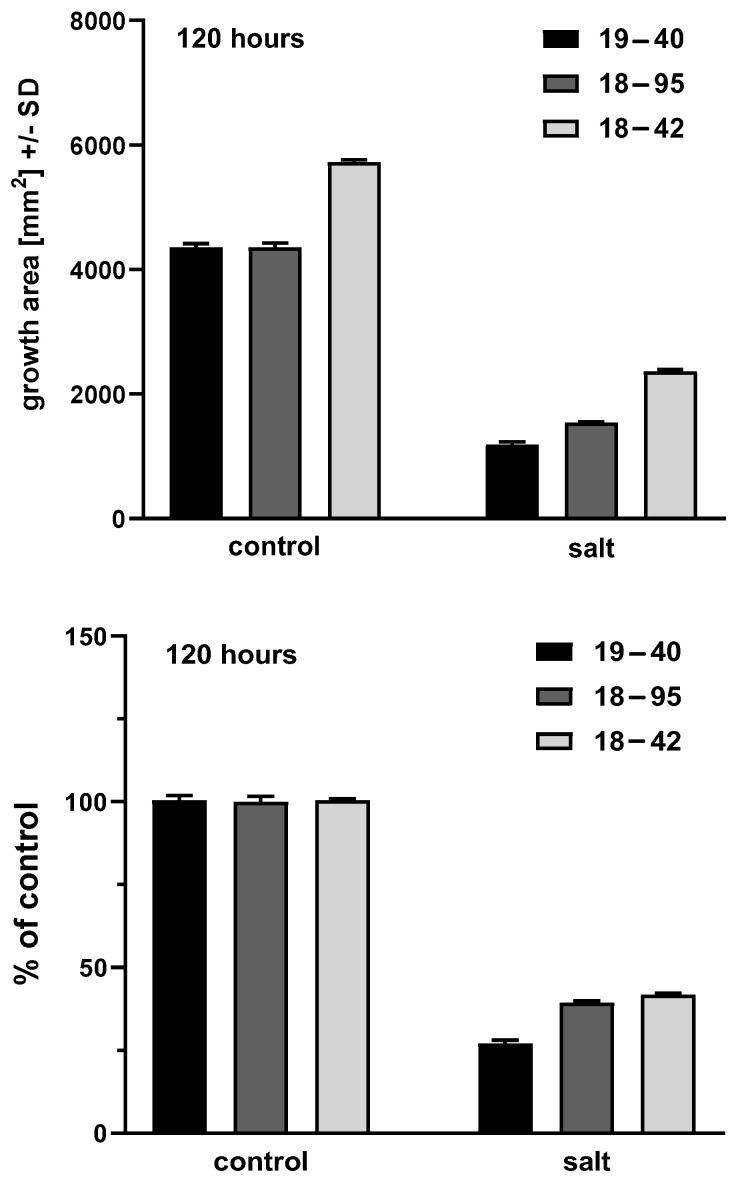
Study of re-infected fungus, strain 19–40 (black bars; the shading of the other bars is the same as in Figure 2 and Figure 3). Representative areas at 120 h, four technical replicates for each bar. Top part: growth areas of the virus-infected (18–95), virus-free (18–42), and virus-free re-infected with virus (19–40), isogenic *A. fumigatus* strains. Left 3 bars, control growth (C) on RPMI agar; right 3 bars, growth in the presence of salt. Control growth for 18–42 is superior to 18–95 and 19–40 (*p* < 0.0001); growth in salt is superior for 18–42 compared to 18–95 and 19–40 (*p* < 0.0001). Salt inhibits growth of all 3 strains in comparison to their controls (*p* < 0.0001). Bottom part: growth in salt as percentage of control growth (control growth considered as 100%). At this time 18–42 growth in salt is superior to 18–59 and 19–40, not only in absolute terms (top part), but also as a percentage of control growth (*p* = 0.008 and *p* < 0.0001, respectively).

As indicated previously, virus-free strain 18–42 had been re-infected with the virus, producing infected strain 19–40 [2,3]. An experiment was performed to validate the experiments discussed above, with measurements at 48–120 h as before. Infected strain 19–40 behaved at all time points as had infected strain 18–95, as follows. Its growth was significantly impaired by salt at all times (*p* < 0.0001). Its growth in the absence of salt was inferior to the virus-free strain 18–42 at all times (*p* = 0.0002–<0.0001). Its growth in the presence of salt was impaired compared to virus-free strain 18–42 at all times (*p* ≤ 0.0001). Relative to control growth, at all times its salt impairment was also greater than the virus-free strain 18–42 (*p* = 0.002–<0.0001). In all these comparisons, the re-infected strain 19–40 was also impaired in growth more, in the presence and absence of salt, and also relative to its control growth, to infected strain 18–95, suggesting even greater impairment by the virus in the newly infected strain 19–40. Figure 4 shows the illustrative 120 h time point data.

## 4. Discussion

Salt stress significantly impairs the growth of the virus-free and virus-infected *Aspergillus* strains. Virus-free strain control growth always significantly exceeded that of the virus-infected, and virus-free strain growth in salt exceeded that of the virus-infected strains. Since virus-free strain growth exceeds that of the virus-infected in the presence and absence of salt, we also examined growth in salt as a percentage of control (absent salt) growth. Initially, as a percentage of control, virus-infected strain growth exceeded virus-free, but at 120 h the virus-free strain growth began to exceed that of the virus-infected strain consistently and significantly, even by this measure as well, and persisted; thus, at that time, the growth of the virus-free strain in salt surged relative to its control growth compared to the virus-infected, or, alternatively, its growth in salt persisted compared to the relative inhibition of the virus-infected strain. The overall greater resistance to salt stress in the virus-free strain may suggest that the virus has a role in fungal survival in nature, in water and soil, where there can be such stresses.

A study with a closely related virus in the *Polymycoviridae* family, with a sequence similarity to our AfuPmV-1, has been published [7]. The virus-infected host had similarly decreased growth and decreased resistance to osmotic stress, compared to a virus-free version, as we have noted here. These authors reported that the virus-infected fungus was also less virulent in mice [7], whereas our virus-infected strain was more virulent in the wax moth infection model [8]. They noted evidence that the virus-infected strain might be impaired in gliotoxin production, in contrast to our report of enhanced gliotoxin production with our AfuPmV-1-infected strain [9], though they noted that the increased production of other *Aspergillus* toxins was associated with virus infection, as we also noted [9]. Their virus-free strain appeared to show consistently increased pigmentation compared to the infected strain, the opposite of what we noted [5].

In conclusion, virus-free strain growth in several control media exceeded virus-infected strain growth, as described here on RPMI-1640 agar, and in other media studied, including liquid mineral medium M9 [5] and Czapek-Dox medium (unpublished studies), although our prior studies indicated no consistent differences in oxidative metabolism assays (XTT assay) in liquid media [4]. Virus-free strain growth in the presence of high salt was always superior to virus-infected strain growth. Thus, infection with this virus impairs the response of *A. fumigatus* to several different stresses, including iron restriction, exposure to *P. aeruginosa* volatiles [4,5], inhibition of cell wall chitin synthesis [10] and high salt osmotic stress. It is possible that salt stress and iron metabolism may be linked, as that has been demonstrated in prokaryotes [11].

We have reported elsewhere on virus-free and virus-infected strain differences in the production of siderophores [5] and, recently, gliotoxin [9]. The observation of differences in siderophore production [5] explains the prior observation of virus-related impairment during iron restriction [4]. That there is virus-related impairment to other stresses (volatiles, cell wall synthesis inhibition, salt) leads to a hypothesis that either there are multiple gene functions affected by the virus, or there is viral impairment of a master, e.g., transcriptional, gene. As the present study showed, temporal assays to document the whole picture of differences resulting from virus infection have been required, here and in the siderophore studies [5], and will be in future studies of stresses and the mechanisms involved.

## Data Availability

Data supporting the reported results are available from the corresponding author.
